# Effects of sliding liquefaction on homogeneous loess landslides in western China

**DOI:** 10.1038/s41598-021-91411-z

**Published:** 2021-06-07

**Authors:** Yong Hong, Xianzhang Ling, Keqiang He

**Affiliations:** 1 School of Civil Engineering, Qingdao University of Technology, Fushun Road No. 11, Qingdao, 266033 Shandong Province China; 2 Cooperative Innovation Center of Engineering Construction and Safety in Shandong Blue Economic Zone, Fushun Road No. 11, Qingdao, 266033 Shandong Province China

**Keywords:** Natural hazards, Geology

## Abstract

Sliding liquefaction is considered to be the cause of high-speed and long-distance sliding of some homogeneous loess landslides in western China. However, there is still a lack of necessary experimental research and analysis on the effects of sliding liquefaction on these landslides. In this work, the effects of sliding liquefaction on irrigation-induced, high-speed and long-distance loess landslides on the South Jingyang Tableland area in China are studied by performing large-scale ring shear tests and using the sled mode. The results are as follows. (1) There are two kinds of long-runout sliding modes of loess landslides on the South Jingyang Tableland: sliding along the terrace surface and sliding within the saturated terrace alluvium, which is associated with sliding liquefaction. Both sliding modes can lead to long-runout sliding. (2) There are some differences in the inclination of the sliding surface between the two sliding modes. Based on the inclination of the sliding surface, the corresponding sliding mode can be distinguished. (3) Under the two sliding modes, the large shear mechanical properties of the two-layer soil composed of loess and alluvial sandy silt show significant differences. The friction between the loess and dry terrace alluvium increases with increasing normal stress and shear rate, while the friction between the loess and saturated terrace alluvium presents the opposite trend. The results show that the sliding distances under different sliding modes present opposite trends with the change in sliding speed. (4) Based on the test results from the ring shear tests and the morphological characteristics of the sliding surface, the sliding mode and sliding distance of a loess landslide can be identified and predicted.

## Introduction

Loess landslides are common geological disasters in the loess area of western China. Loess landslides can be generally divided into four basic types (homogeneous loess landslides, loess interface landslides, loess mudstone-layer-plane landslides, and loess mudstone-cutting-layer landslides), in which homogeneous loess landslides occur most frequently among all loess landslides with the widest distribution range, sudden occurrence, long-sliding distance, and severe disaster^1^.

Large-scale homogeneous loess landslides occur frequently along the edge of the South Jingyang Tableland, Shaanxi Province, China. At present, the types, development characteristics and formation mechanisms of loess landslides on the South Jingyang Tableland have been comprehensively studied by performing field investigations and laboratory tests^2–6^. Studies have shown that the high and steep slopes, special physical and mechanical properties of the loess and continuous rise in groundwater level are the main factors of loess landslide formation in this area^7–10^. Irrigation-water infiltration-induced seepage is considered to be the major contributor to the triggering of loess landslides ^11^. The main mechanism is long-term water diversion irrigation on the top area of the tableland that causes a sustained rise in groundwater levels, thus increasing water content and reducing shear strength in the loess, which eventually leads to the occurrence of loess landslides.

The loess landslides that have occurred on the South Jingyang Tableland can be categorized into two types, namely, loess flowslides and loess slides^12,13^. Loess flowslides behave in a quasi-liquid state that can move a very long distance at a high velocity, while loess slides behave in a quasi-plastic state and move a shorter distance^12^. The loess landslides surveyed on the South Jingyang Tableland exhibited strong interactions between landslide materials originating from the edge of the slope and the terrace during landsliding^13^. The sand outburst phenomenon and surface-sand boiling on site were found to be evidence of the fluidisation of soil, also referred to as sliding liquefaction^14^. Relevant research also confirmed that terrace sediments easily show inducible high-pore water pressure under undrained conditions and have high susceptibility to liquefaction^11,13^. Sliding liquefaction at the interface of the landslide deposit and terrace sediments during loess flowslide is considered to be the main cause of high-speed long-runout loess sliding in this area^8,10,14^. However, the influence of this kind of liquefaction on the sliding of loess landslides has been underexplored in the literature.

In fact, the occurrence and sliding of loess landslides are large shear deformation processes of sliding masses along certain sliding paths. To reveal the mechanisms of rapid and long-travel landslides, it is essential to observe the behaviours of shear zones during shearing^15^. In this study, during the kinetic process of landsliding, the interactions between slope deposits and substrates involve soils in different parts of the sliding paths that have different physical and mechanical properties (i.e., loess deposits and terrace sediments). Research on the contact shear action between different soils is the key link that reveals the influence of sliding liquefaction on landslide movement. At present, research on the large shear mechanical properties of soil in the process of landsliding is mainly aimed at a single soil, but there is a lack of relevant experimental research on the contact shear mechanical properties between two kinds of soil with different physical and mechanical properties during sliding. Because of the lack of laboratory tests of soil shear mechanical properties and calculation models of sliding distance, interpretations of the effect of sliding liquefaction on loess landslides on the South Jingyang Tableland are still not available.

In this paper, the frequent homogeneous loess landslides on the South Jingyang Tableland are utilized as the research object. The effect of sliding liquefaction on high-speed and long-distance loess landslides in this area is studied by large-scale ring shear tests and a sled mode.

## Overview of study area

**Figure 1:**
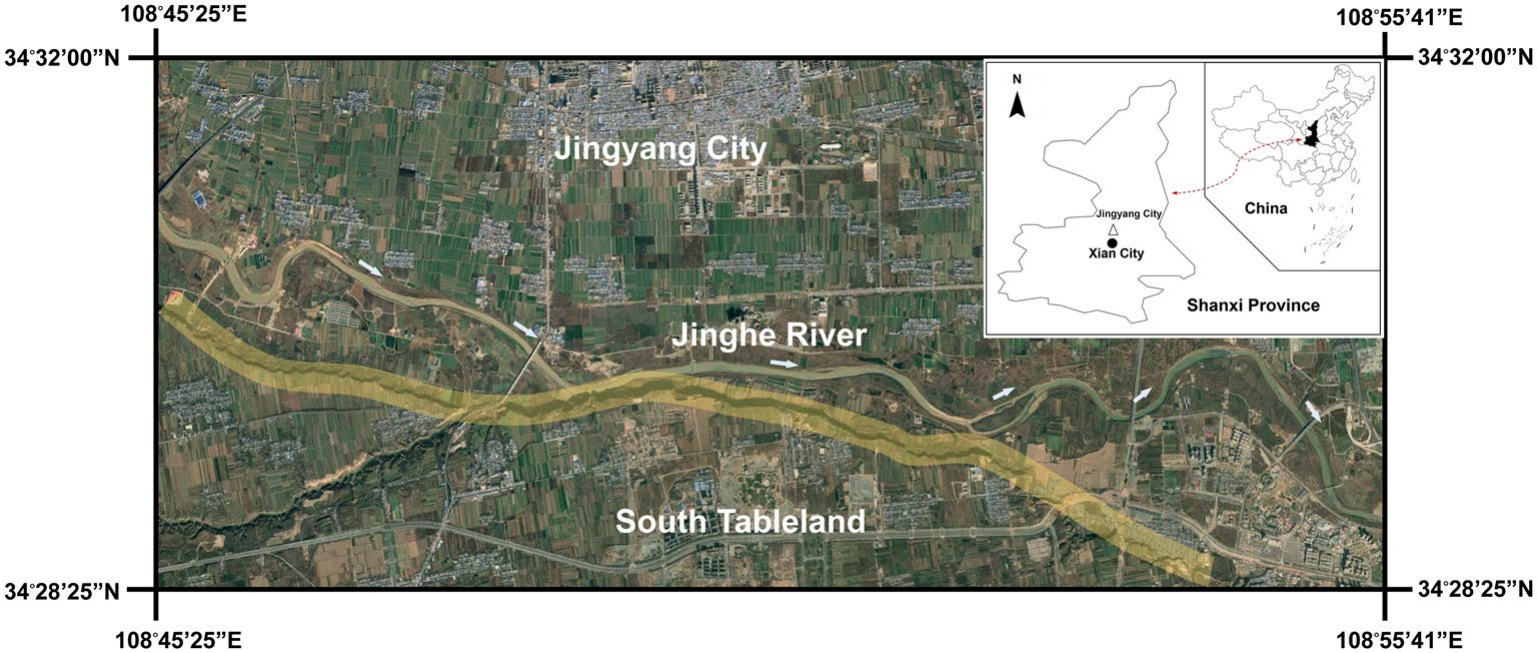
Location of the South Jingyang Tableland (drawing based on Google Earth).

The South Jingyang Tableland is located on the south bank of the Jinghe River, Jingyang County, Shaanxi Province, China, with an area of approximately 70 km^2^ and a length of 27.1 km from east to the west (as shown in Fig. 1). The top of the tableland is used for agricultural cultivation. The slope toe of the tableland edge is directly connected to the open and flat Jinghe River terrace. Due to the strong lateral erosion of the Jinghe River, a steep slope with a height of 50–90 m and a slope of 40°–80° has formed at the edge of the tableland.

**Figure 2:**
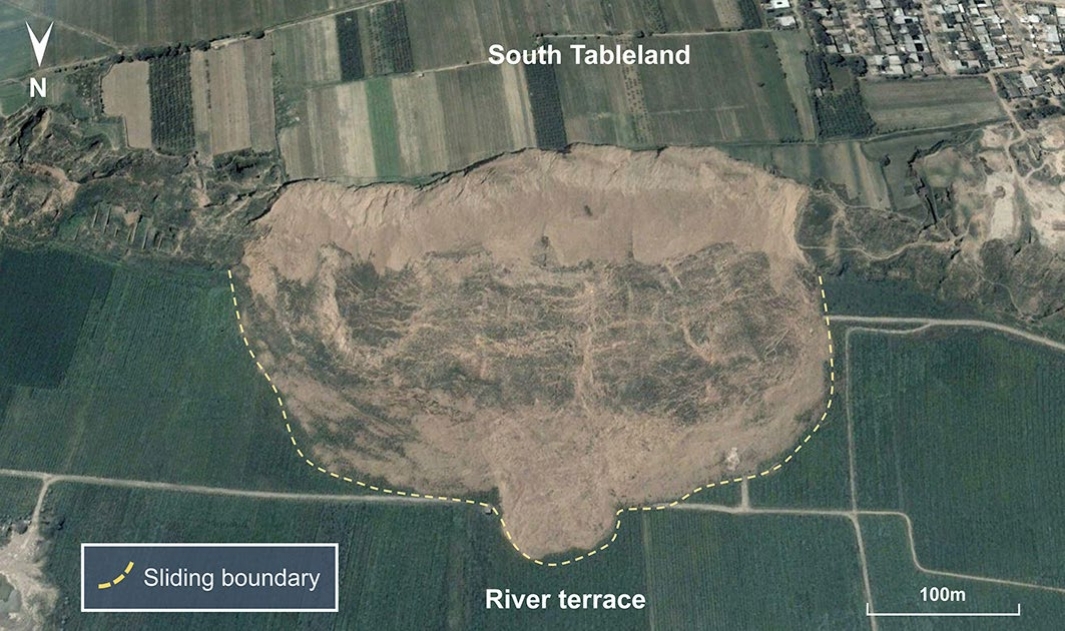
Morphology of a landslide after sliding near Dongfeng Village in July 2003. The sliding mass presents a flow-sliding state on the terrace and is spread across the terrace in a semicircular or circular shape.

With the large-scale use of the Jinghe River for agricultural irrigation in the South Jingyang Tableland area, landslides occur frequently and are distributed intensively along the edge of the tableland. At least 27 large loess landslides have occurred^6^. The landslide area is shown with yellow shading in Fig. 1. The landslides in this area share many characteristics, such as a short incubation period, suddenness, fast-sliding speed, long-sliding distance and high frequency, which are typical of high-speed and long-distance loess landslides. The volume of landslide mass in this area is large, mostly between tens of thousands of cubic metres and more than one million cubic metres. The sliding mass presents a flow-sliding state on the terrace and is spread across the terrace in a semicircular or circular shape (Fig. 2). The sliding distance is generally 100–300 m but can even reach 400 m.

**Figure 3:**
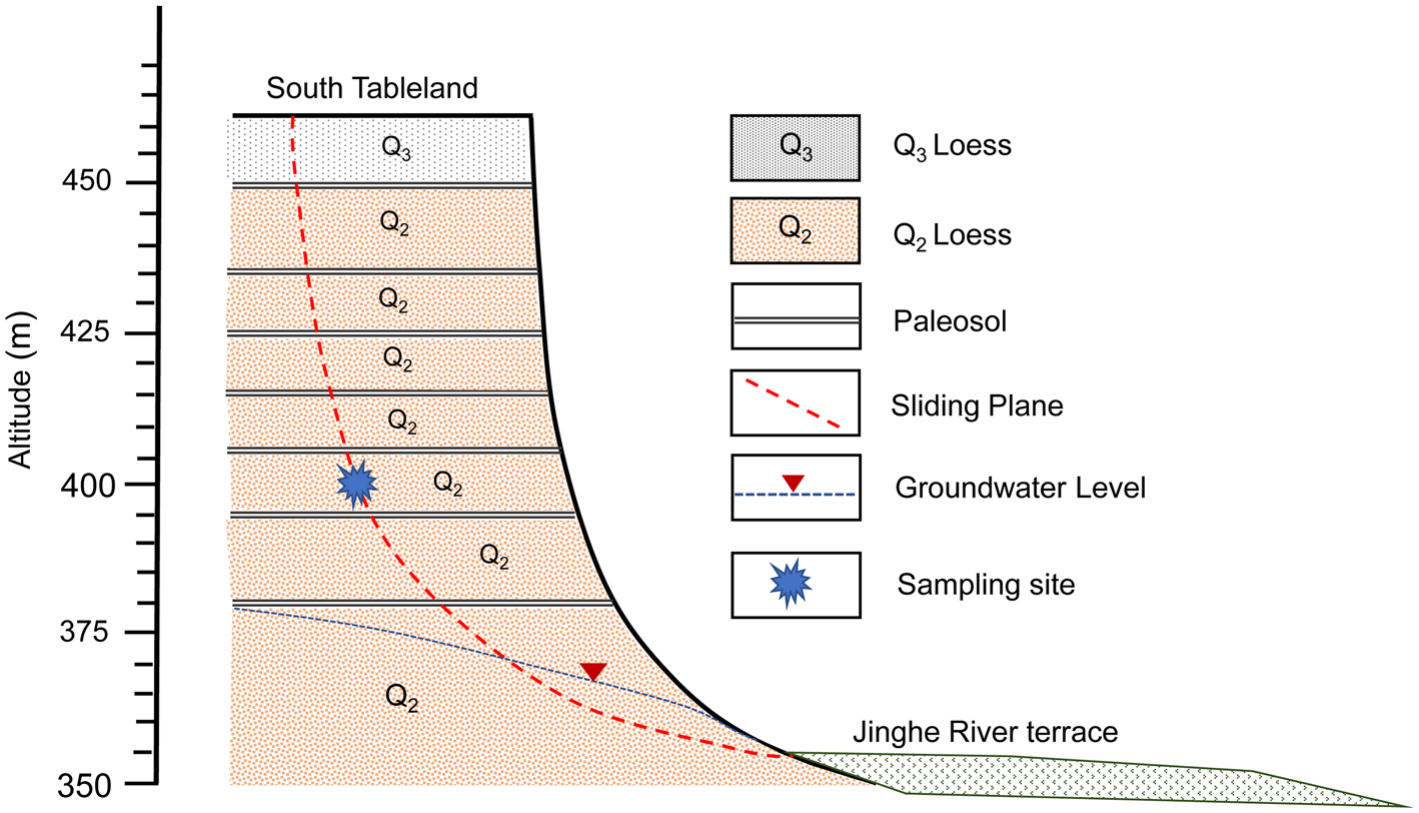
Stratigraphic profile of the loess landslide near Dongfeng Village on the South Jingyang Tableland.

The loess landslide shown in Fig. 3 is mainly composed of thickly layered, Cenozoic middle late Pleistocene aeolian loess, with several layers of reddish brown paleosol. The upper stratum of the slope consists of late Pleistocene aeolian Q_3_ loess. The Q_3_ loess layer has an average thickness of 10 m. The paleosol layer in the loess stratum is relatively thin, with an average thickness of approximately 0.2–0.3 m. The Q_2_ loess layer of middle Pleistocene age (with a thickness greater than 50 m) constitutes the main body of the landslide, and its mechanical properties directly affects the stability of the slope. According to the field survey, sliding surfaces of loess landslides are mainly located in the thick Q_2_ loess layer. Due to the large thickness of strata, the burial depth of the potential sliding surface in the Q_2_ loess stratum is deep, and the soil moisture content there is low. Based on the composition of the slide mass and the situation of the failure plane, this loess landslide is recognized as a homogeneous loess landslide, namely, landsliding within the loess^16^.

**Figure 4:**
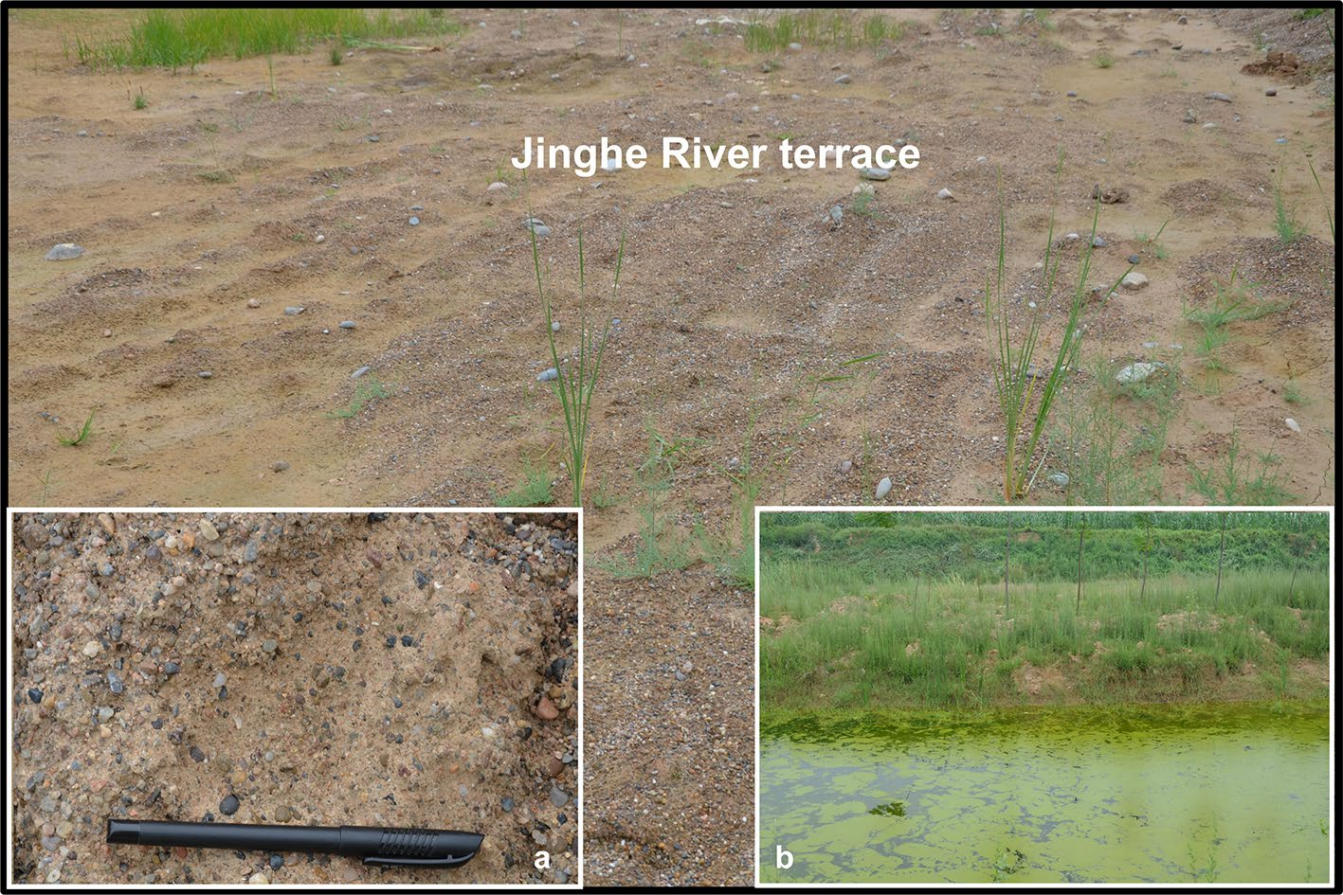
Photograph of the Jinghe River terrace: (**a**) alluvial deposits in the terrace; and (**b**) shallow groundwater at the surface in the terrace.

The Jinghe River terrace is mainly composed of alluvial deposits (Fig. 4). The upper part of the terrace alluvium is mainly composed of sand and silt, which is well graded (Fig. 4a), and the lower layer is a pebble layer with larger particle sizes.

The ground water level in the Jing River terrace is high^4,5^ (Fig. 4b). The soil moisture content of the alluvium above the groundwater level is low, and the terrace soil layer below the groundwater level is in a saturated state.

**Figure 5:**
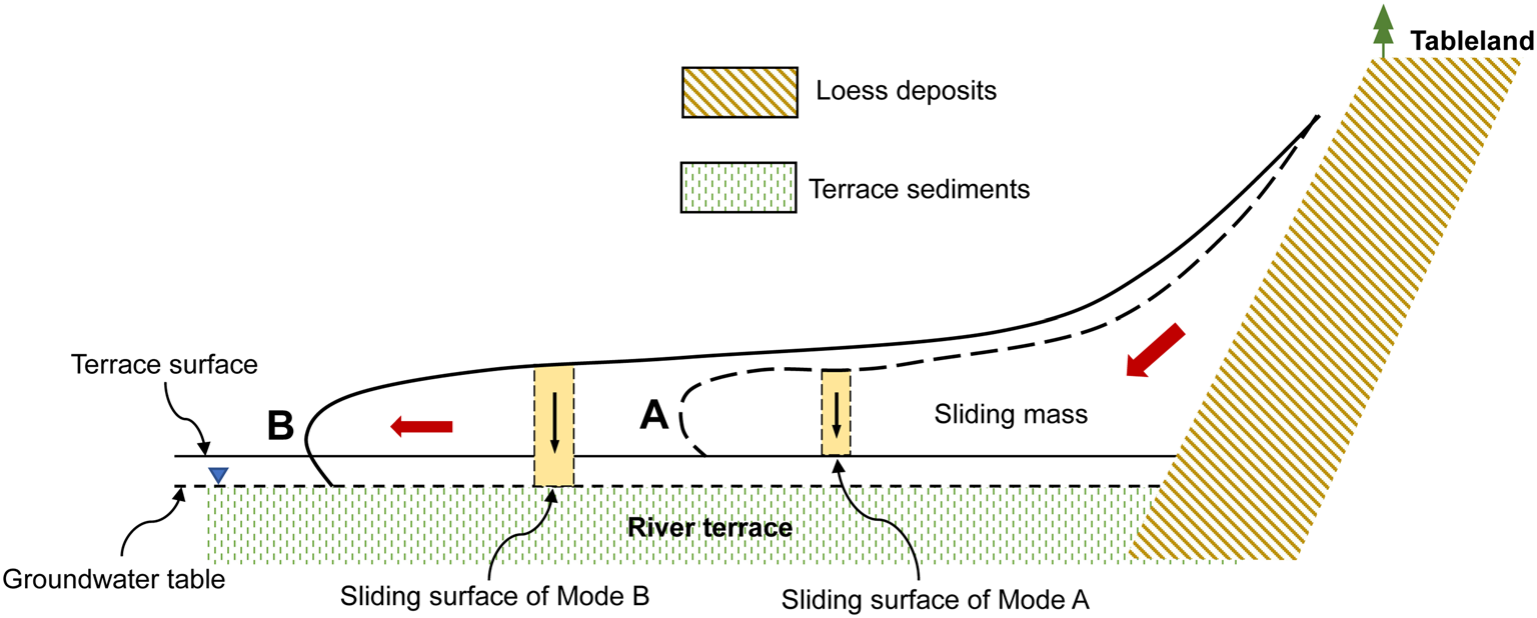
Sliding modes of the loess-sliding mass along the Jinghe River terrace. (**A**) The loess-sliding mass only slides along the terrace surface. (**b**) The loess-sliding mass slides along the saturated terrace stratum below the groundwater level.

According to the different water-bearing states of the soil in the terrace stratum, the possible contact relationship between the loess-sliding mass and the terrace stratum during sliding can be divided into two modes (Fig. 5). Mode A: the loess-sliding mass only slides along the terrace surface. The sliding surface lies between the loess and the relatively dry terrace sediment. Mode B: the loess-sliding mass slides along the saturated terrace stratum below the groundwater level. With the sliding of the sliding mass on the terrace, a high-speed shear environment is formed between the bottom of the sliding mass and the soil layer of the terrace. This study simulates the closed undrained, fast shear environment at the sliding surface with ring shear testing and studies the contact shear mechanical action between loess and different water-bearing terrace soils (loess and dry terrace sediment (LDT) and loess and saturated terrace sediment (LST)) during the sliding process.

## Ring shear test implementation

### Test specimens

**Figure 6:**
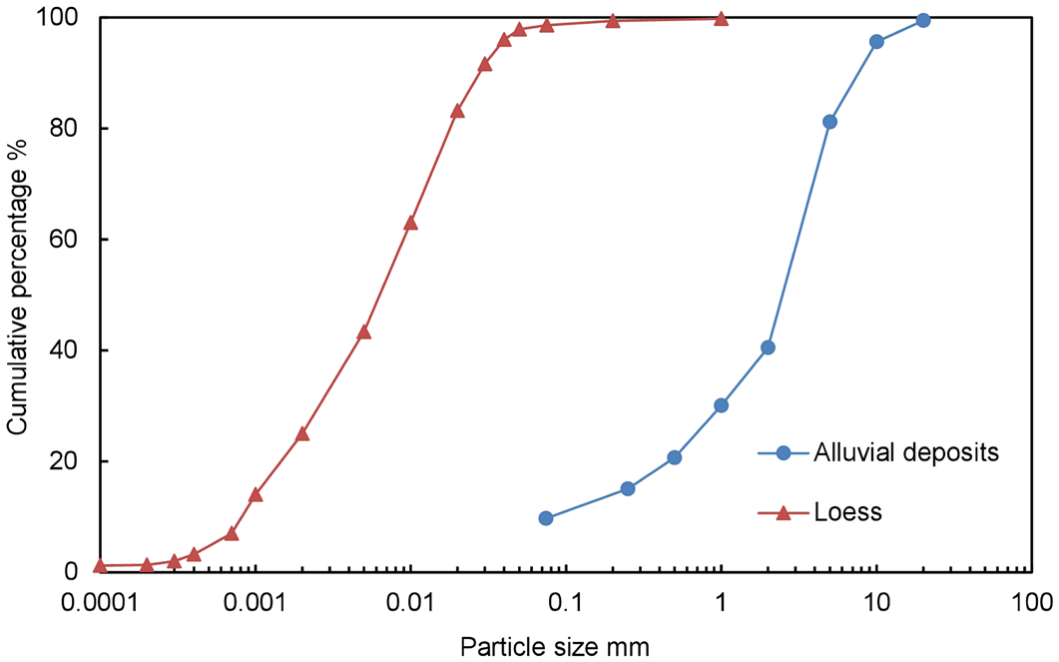
Gradation curves of Q_2_ loess and terrace alluvial soil.

In this study, loess samples collected from the Q_2_ loess stratum in the middle of the sliding surface (Fig. 3) and two-layered soil composed of loess and terrace soil were tested. Q_2_ loess is categorized as silty clay, which is characterized by greyish yellow, dense, fine particles, a uniform texture and a high viscosity. The basic physical properties of undisturbed Q_2_ loess are shown in Table 1. The terrace alluvial soil is mainly composed of silt and sand, with a clay content of 9%, silt content of 54% and fine-sand content of 36%, which is categorized as sandy silt. Figure 6 provides the grading curves of Q_2_ loess and terrace alluvial soil.

**Table 1:** Physical mechanical properties of the loess samples.

Specific gravity	Water content (%)	Density (g/cm^3^)	Void ratio	Liquid limit (%)	Plastic limit (%)	Collapsibility coefficient	Compression coefficient (/MPa)
2.71	6.3	1.78	0.62	29.16	18.25	0.03	0.16

### Test equipment

**Figure 7:**
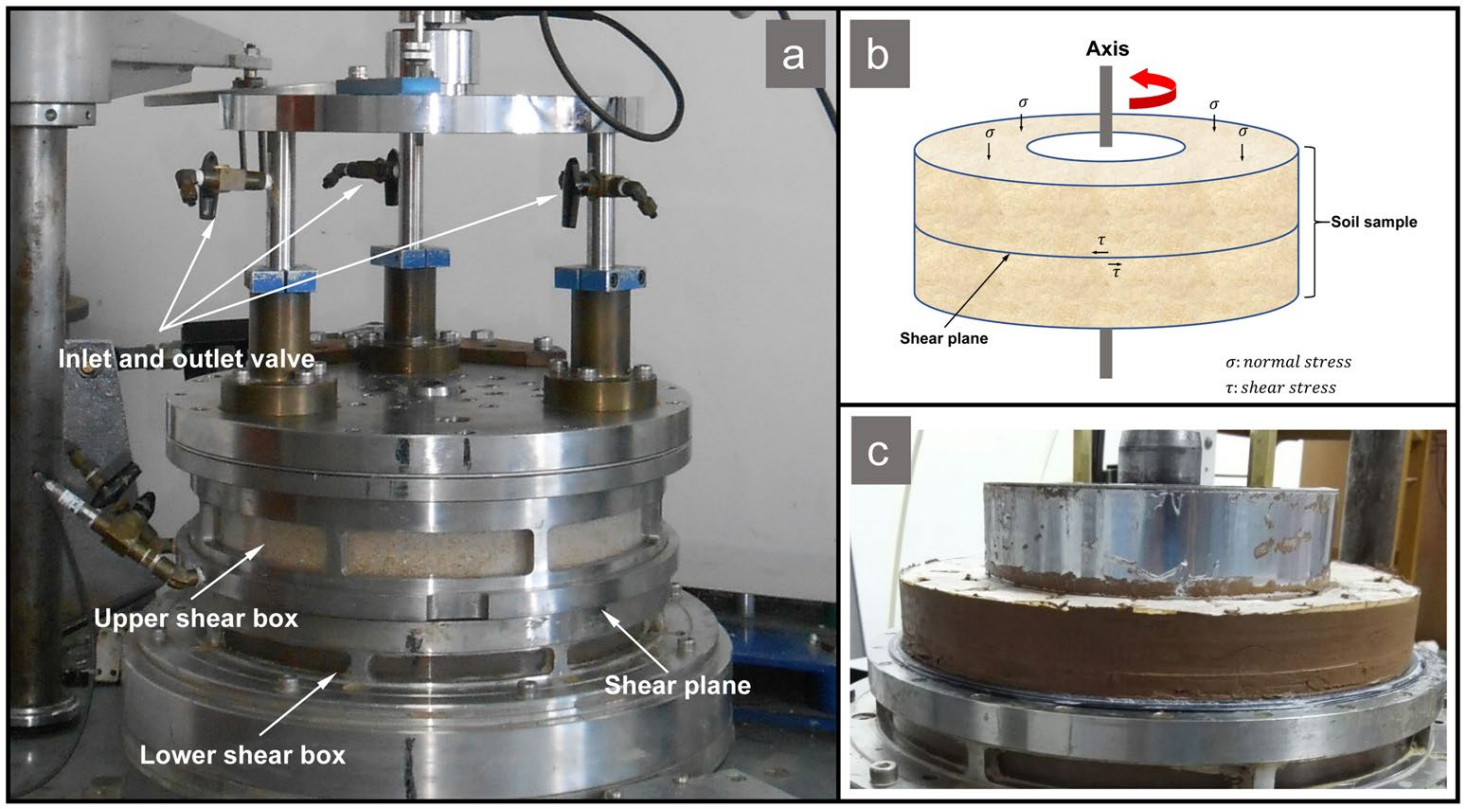
Ring shear apparatus. (**a**) shear box; (**b**) stress state of soil in the shear box; and (**c**) exposed soil after removal of upper shear ring.

The ring shear apparatus is most appropriate in the study of long-travel landslide mechanisms because there is no limitation for shear displacement, and the equipment can provide higher normal stresses corresponding to real landslides. The test equipment used in this research is a DPRI-3, large-scale, high-speed, high-pressure, ring shear apparatus developed by the Disaster Prevention Research Institute of Kyoto, University of Japan (Fig. 7a). This ring shear apparatus consists of a disconnect-type annular shear box, loading system, monitoring system, gap control, and shear rate control system. Figure 7b,c presents the soil state in the ring shear box and the exposed soil sample. The soil sample in the ring-shear box is laterally confined between pairs of doughnut-shaped upper and lower confining rings, and the landslide is simulated by applying the normal stress and shear stress that exist at the sliding surface in the field. The inner diameter of the shear box is 21.0 cm, the outer diameter is 31.0 cm, the shear area is 408.4 cm^2^, and the maximum loading height is 10 cm. The maximum vertical loading capacity of the ring shear apparatus can reach 500 kPa. The use of precision machining technology and servo control systems can ensure the implementation and conversion of drained and undrained test conditions in the shear test, and there is no leakage problem, which ensures the accuracy and authenticity of the test results. Further detailed information on the design and construction of the ring shear apparatus, as well as the operation method, can be found in the literature^17–23^.

### Test programme

In this study, undrained ring shear tests were conducted under different normal stresses and shear rates as follows.

#### Ring shear testing of loess

First, the Q_2_ loess collected from the site was crushed and then dried in an oven at 105 ℃ for 24 h. After the sample was completely dried, it was further crushed, and then the soil sample was completely cooled. During sample loading, the dry loess was poured into the ring shear test box in layers and tamped with a wooden hammer, and then the predetermined vertical load was applied.

During the test, the dry loess was consolidated under the predetermined normal stress until the vertical settlement of the soil sample in the shear test box remained unchanged. Then, the shear test was performed for each of the specimens with the same normal stress (σ) at the predetermined rate, and the shear test was stopped when the stable residual shear strength was reached. Considering the thickness of the landslide mass in the actual landslide and the loading capacity of the ring shear apparatus, normal stresses of 100 kPa, 200 kPa and 300 kPa and shear rates of 0.01 cm/s (r′), 0.10 cm/s (r′) and 1.00 cm/s (r′) were selected for this study. The dry loess tests were conducted in nine groups (see Table 2 for the test groups). The same normal stress and shear rate were used in the following tests.

**Table 2:** Test grouping of the Loess, LDT and LST.

*σ* (kPa)	*r′* (0.01 cm/s)	*r′* (0. 1 cm/s)	*r′* (1.0 cm/s)
100	Loess 1; LDT 1; LST 1	Loess 2; LDT 2; LST 2	Loess 3; LDT 3; LST 3
200	Loess 4; LDT 4; LST 4	Loess 5; LDT 5; LST 5	Loess 6; LDT 6; LST 6
300	Loess 7; LDT 7; LST 7	Loess 8; LDT 8; LST 8	Loess 9; LDT 9; LST 9

#### Ring shear testing of two-layered soil: loess and dry terrace alluvial soil (LDT)

After a ring shear test was performed on a dry loess sample, the upper cover of the shear box and the outer shear ring of the upper part of the shear test box were removed, and the soil above the shear plane was removed, but the soil in the lower shear box was retained; then, the rubber ring at the contact position of the upper and lower shear ring was cleaned with acetone and coated with sealing silicone grease, and the upper outer shear ring was reinstalled and fixed with the guide rod.

The dried terrace alluvial soil was loaded into the shear box in layers and compacted with a wooden hammer. Then, the two-layer soil sample composed of upper dry terrace soil and lower loess was reapplied with a predetermined vertical load for consolidation until the vertical settlement of the soil sample in the shear test box remained unchanged. Then, the shear test was carried out according to the predetermined normal stress and shear rate (see Table 2 for the test groupings).

#### Ring shear testing of two-layered soil: loess and saturated terrace alluvial soil (LST)

First, the samples were loaded and consolidated in the same way as in the loess and dry terrace alluvial soil tests. The samples in the shear test box were composed of upper dry terrace alluvial soil and lower dry loess. Then, deionized water was injected into the terrace soil in the shear box through the upper water inlet pipe of the shear box, and the valve of the drainage device on the upper part of the shear box was opened for drainage (Fig. 7a). When no bubbles were observed in the upper drainage pipe and the flow velocity was stable, the terrace alluvial soil was determined to be in a saturated state. The drainage valve of the ring shear test box was closed to place the soil in the shear test box into a completely undrained state. Finally, the soil samples prepared according to the above methods were tested under the predetermined normal stress and shear rate (see Table 2 for the specific groupings).

### Test results

**Figure 8:**
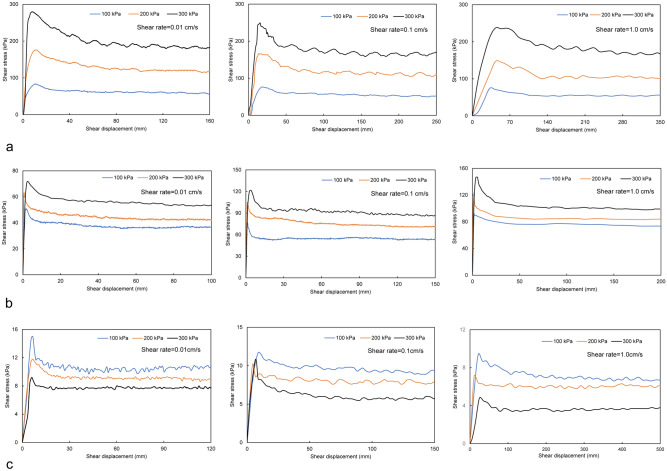
Change in the shear stress of the Q_2_ loess, LDT and LST under large shear deformation conditions under different shear rates: (**a**) Q_2_ loess; (**b**) LDT; and (**c**) LST.

The Fig. 8 shows the curves of shear stress and shear displacement of the Q_2_ loess, LDT and LST. Compared with the shear stress of loess, the shear stress of the LDT and LST measured by this ring shear test is actually the friction resistance produced by the interaction of the two soils with different properties and states along the preset shear plane, rather than the internal shear force of the soil.

## Analysis and discussion

At present, research on the mechanisms of loess landslides on the South Jingyang Tableland shows that the high and steep slopes, open free surfaces, unique physical and mechanical properties of the loess and rising groundwater levels are the main factors for the formation of landslides. The large thickness of the loess layer and the steep slope of the tableland directly connected to the terrace provide favourable geological and topographic conditions for high-speed and long-distance sliding. The sliding process can be divided into two stages: (1) sliding along the slope and (2) sliding on the terrace.

The first stage reflects the sliding process of the loess mass along the sliding surface in the slope under the action of gravity (a continuous sliding surface is formed in the slope). This stage spans the starting and accelerating processes of the sliding mass on the slope.

**Figure 9:**
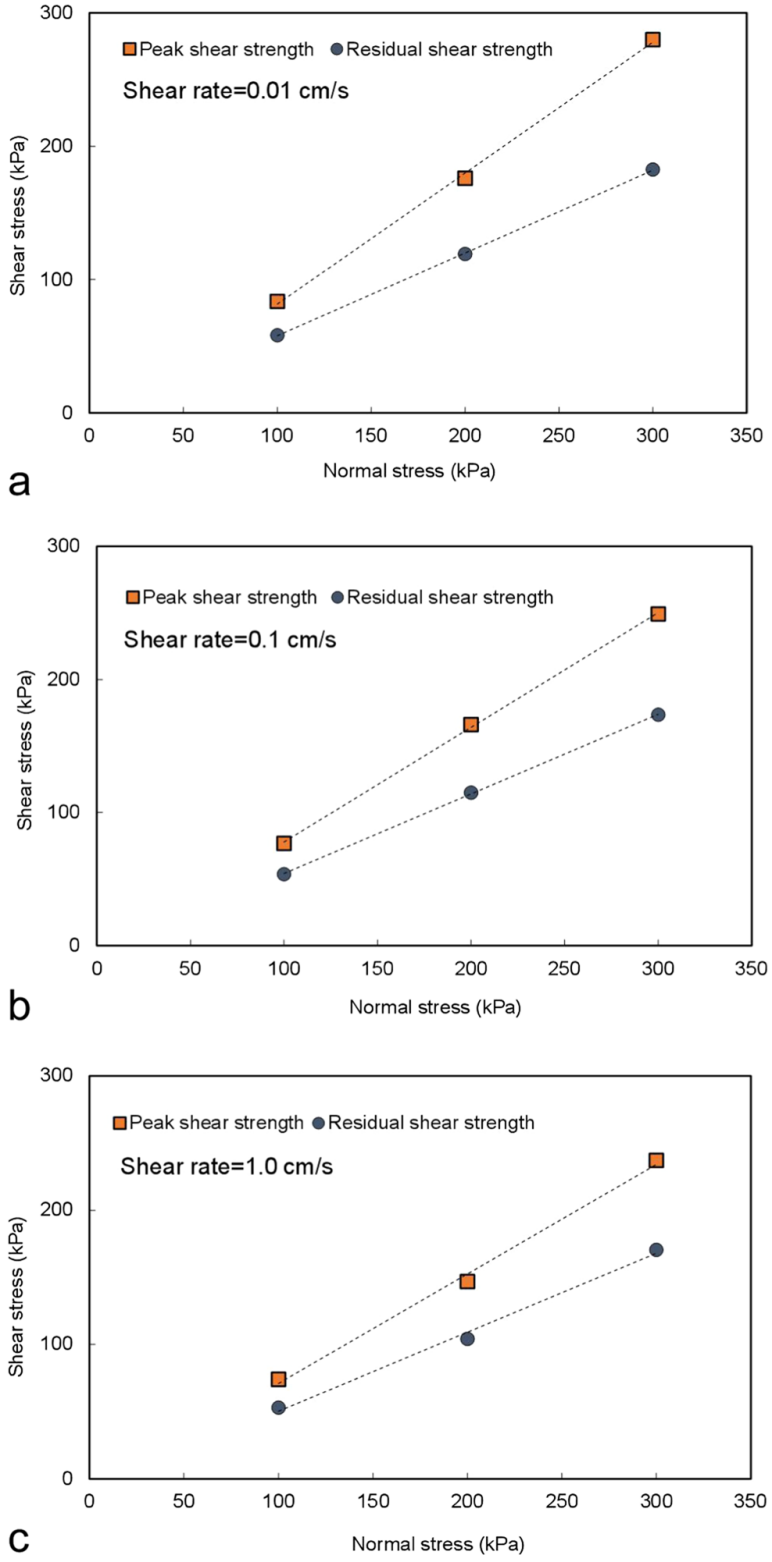
Relationship between the shear strength (peak shear strength and residual shear strength) and normal stress of the dry Q_2_ loess.

Figure 9 shows the relationship between the shear strength (peak shear strength and residual shear strength) and normal stress of the dry Q_2_ loess measured by the ring shear test. The Q_2_ loess has a high peak shear strength in the dry state, which makes the dry Q_2_ loess stratum resistant to slope instability and failure; therefore, this part of the loess stratum constitutes the locking section of the slope. However, the dry Q_2_ loess has obvious mechanical characteristics of strain softening in the process of large shear deformation; that is, there is a large strength difference between the peak shear strength and residual shear strength. Therefore, at the beginning of the landslide failure stage, due to the sharp decrease in the shear strength of the loess, the anti-sliding force in the loess stratum will decrease greatly, which will lead to the sliding mass obtaining great sliding force and the rapid release of a considerable amount of potential energy. This will result in a high acceleration during the initial sliding of the landslide and will provide a high initial starting speed and kinetic energy for the subsequent sliding, which is the main reason for the high initial sliding speed of the loess landslide. The sliding that occurs during this stage is a sudden start-up and acceleration process, which shows that the loess-sliding mass slides rapidly along the sliding surface at a fairly high speed under the action of a large sliding force.

In the second stage, the sliding mass slides out of the slope toe at a very high speed, rushes into the open Jinghe River terrace and covers the terrace stratum. A strong interaction occurs between the sliding mass and terrace within a short time, including vertical loading and horizontal shearing. Thus, the long-distance movement of landslides is closely related to the contact relationship and mechanical action between the loess-sliding mass and terrace strata.

As mentioned above, there are two possible sliding modes between the loess mass and terrace strata.

### Mode A

**Figure 10:**
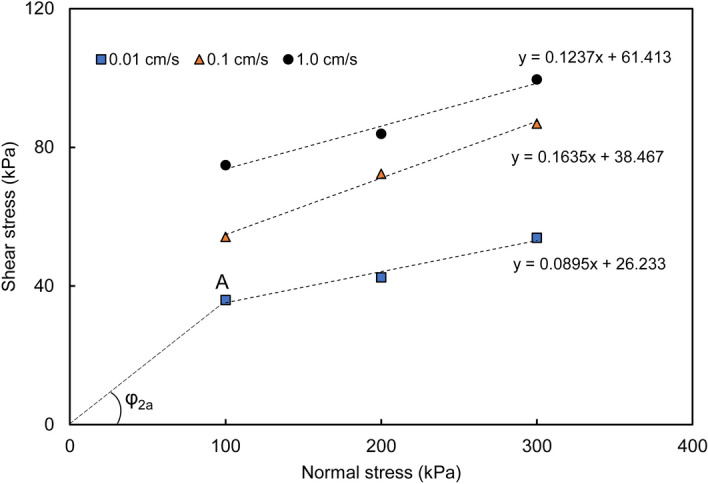
Relationship between the shear stress and normal stress of the LDT.

The sliding mass slides along the terrace surface. If the impact force of the sliding mass on the terrace is not sufficient to break through the upper alluvial soil layer of the terrace and contact the lower saturated stratum, the sliding mass can only slide along the surface of the terrace. The sliding surface lies between the loess and the dry terrace soil layer. Figure 10 shows the relationship between the shear stress and normal stress of the LDT. As shown in the figure, the shear stress of the LDT increases with increasing shear rate and normal stress.

### Mode B

**Figure 11:**
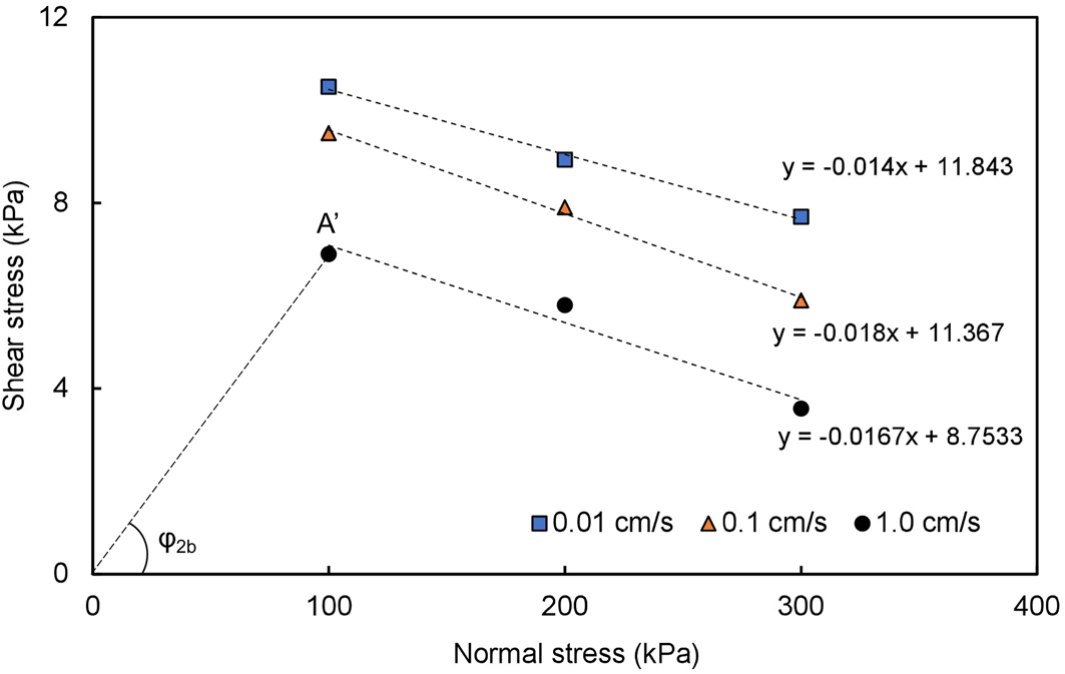
Relationship between the shear stress and normal stress of the LST.

Under the action of fast-moving sliding mass loading, the sliding mass breaks through the upper dry terrace stratum and contacts the lower saturated terrace stratum. The sliding body slides along the saturated terrace soil layer, forming shear conditions with a high sealing degree, fast shear rate and high-pore water pressure at the sliding surface. Figure 11 shows the relationship between the shear stress and normal stress of the LST. As shown in the figure, the shear stress of the LST is not only very small but also decreases with increasing normal stress and shear rate. If the normal stress is large enough, the shear stress of the LST will even decrease to 0, which will lead to the complete loss of sliding resistance at the sliding surface, that is, sliding liquefaction.

The sliding distances of the studied South Jingyang Tableland landslide under the two sliding modes were analysed and compared using the sled model with the test results from the above ring shear tests.

**Figure 12:**
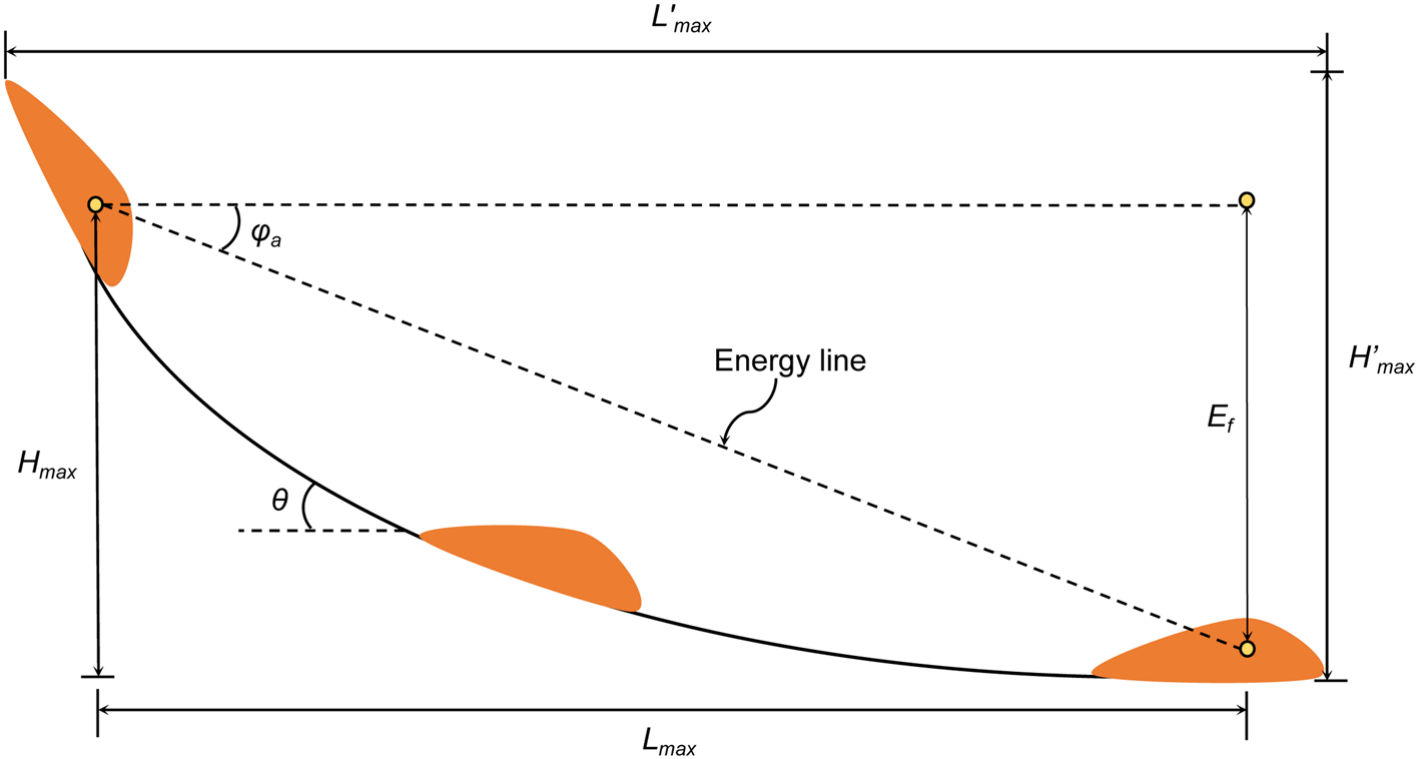
Schematic diagram of the sled model.

Figure 12 shows the schematic diagram of the sled model. In the sled model, it is assumed that all energy loss during landslide movement is caused by friction. The maximum horizontal sliding distance is *L*_*max,*_ and the maximum vertical sliding distance is *H*_*max*_. Then, the work done by the friction resistance in the sliding process *E*_*f*_ is:

1 $$E_{f} = \int\limits_{0}^{{L_{{max}} }} {mg\;\cos \theta \;\tan \varphi _{a} \frac{{dx}}{{\cos \theta }}} = mg\;L_{{max}} \;\tan \varphi _{a}$$

The work done by gravity in the process of sliding *W*_*c*_ is:

2 $${W}_{c}=mg{H}_{max}$$

According to the principle of energy conservation, the following formula is used:

3 $${E}_{f}={W}_{c}$$

According to formulas (1)–(3), formula (4) can be obtained:

4 $$\tan \varphi _{a} = H_{{max}} /L_{{max}}$$

In the formula, *φ*_*a*_ is the apparent friction angle of the sliding body, *θ* is the inclination angle of the sliding surface, *m* is the mass of the sliding mass, and *g* is the acceleration of gravity.

Based on the above formula, when the apparent friction angle *φ*_*a*_ and *H*_*max*_ of the sliding path are known, *L*_*max*_ can be calculated according to formula (4), and the energy line can be drawn (as shown in Fig. 12). Hsu (1978) improved the model by moving the starting point of *H*_*max*_ from the centroid of the sliding body to the top edge of the sliding surface, and the values of *H*_*max*_ and *L*_*max*_ also change accordingly^24^ (as shown in Fig. 12, *H*′_*max*_ and *L*′_*max*_, respectively). This improvement is convenient for the application of the sled model in landslide distance prediction.

The most important parameter in the sled model is *φ*_*a*_, which is equivalent to the inclination of the energy line. It is the apparent friction angle, not the internal friction angle of the soil^25^. After the undrained ring-shear apparatus was developed, *φ*_*a*_ was obtained from formula (5) and the results of ring shear testing^26^

5 $$\tan \varphi _{a} = \frac{{\tau _{{ss}} }}{{\sigma _{0} }}$$

where *τ*_*ss*_ is the measured shear strength at a steady state (kPa) and *σ*_*0*_ is the initial total normal stress (kPa).

According to the apparent friction angles of different sliding parts of the landslide and corresponding energy lines, the approximate sliding distance of the studied South Jingyang Tableland landslide can be estimated. For the loess landslide on the South Jingyang Tableland, the apparent friction angle along the sliding path can be approximately divided into two parts, namely, *φ*_*1*_ in the slope (shear in the case of dry Q_2_ loess) and *φ*_*2*_ generated during sliding along the terrace (undrained shear between loess masses with different water-bearing alluvial deposits). The apparent friction angle mobilized during movement in the slope and alluvial area are measured using the undrained ring shear apparatus.

**Figure 13:**
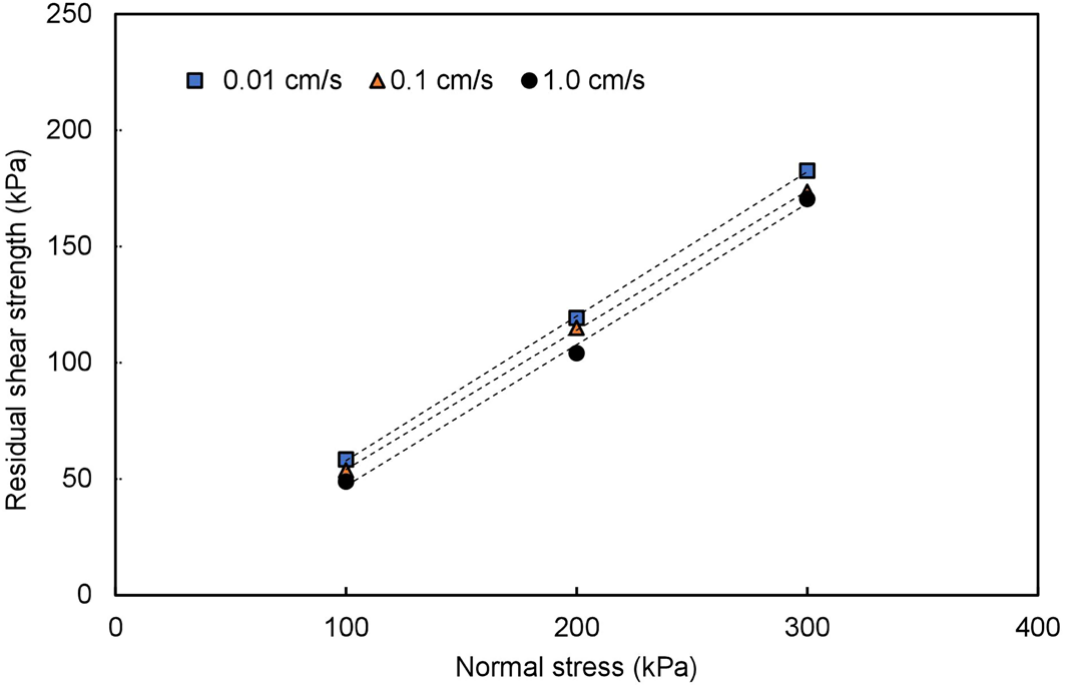
Relationship between the residual shear strength and normal stress of the dry Q_2_ loess under different shear rates.

Figure 13 shows the results of the relationship between the residual shear strength and normal stress of the dry Q_2_ loess under different shear rates. *φ*_*1*_ can be obtained from the residual friction angle of the dry loess. The residual friction angle of the dry Q_2_ loess in the slope is approximately 31° on average. As the shear rate effect is not considerable, the apparent friction angle of dry loess *φ*_*1*_ under different shear rates is approximately equal to its residual friction angle; therefore, *φ*_*1*_ on the slope is taken as 31°.

In the stage of sliding along the terrace, the apparent friction angle *φ*_*2*_ can be obtained by connecting the coordinate origin with the corresponding shear stress values under different normal stresses, as shown in Figs. 10 and 11. The normal stress value is determined according to the thickness of the specific landslide mass. Figure 10 shows an example of the apparent friction angle of LDT (*φ*_*2a*_) obtained at a normal stress of 100 kPa and a shear rate of 0.01 cm/s. From the normal stress value, the corresponding shear stress value (A) is determined. The angle between 0 A and the horizontal axis is the apparent friction angle of the LDT (*φ*_*2a*_) under the corresponding normal stress of 100 kPa and shear rate of 0.01 cm/s. In Fig. 11, the apparent friction angle of LST (*φ*_*2b*_) at a normal stress of 100 kPa and a shear rate of 1.0 cm/s is the angle between 0 A′ and the horizontal axis. The apparent friction angles along the terrace under other normal stresses and shear rates can also be obtained by the above method.

**Figure 14:**
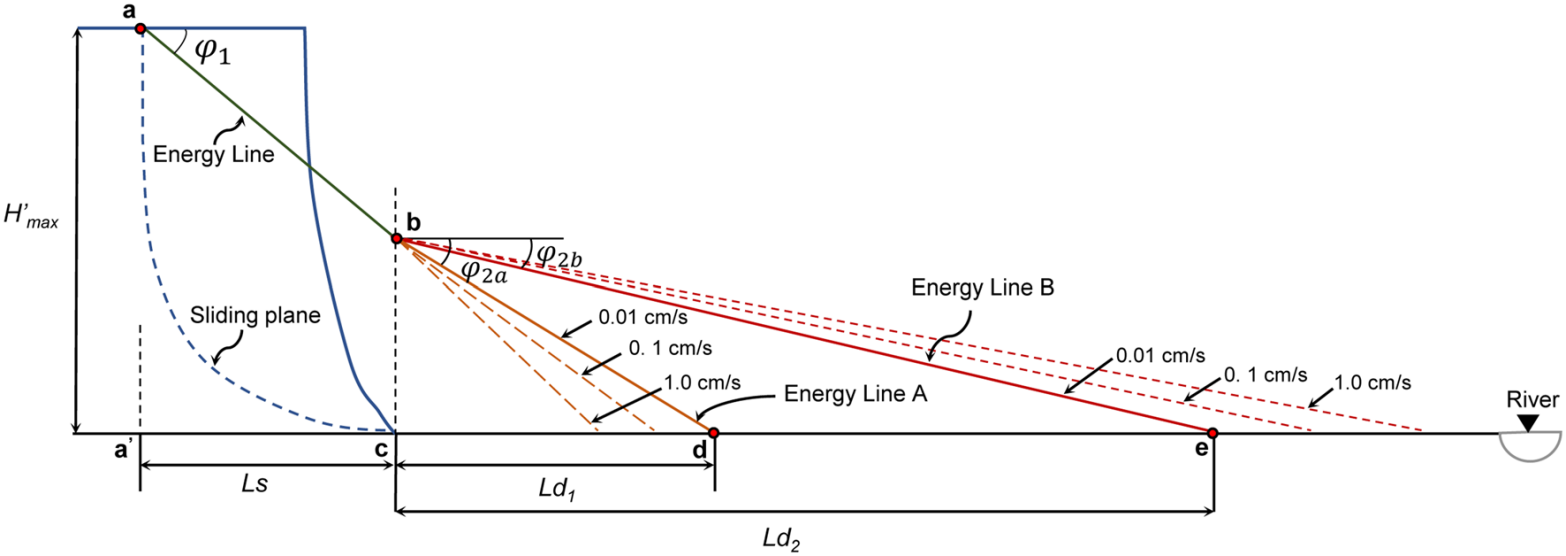
Energy line diagram of the studied South Jingyang Tableland landslide under two sliding modes, A and B.

Figure 14 shows the energy line diagram of the South Jingyang Tableland landslide under two sliding modes, A and B, drawn according to *φ*_*1*_, *φ*_*2a*_, and *φ*_*2b*_. First, the energy line from the vertex of the back edge of the sliding surface (a) at the inclination of *φ*_*1*_ (31°) is drawn to the foot of the slope to obtain point b. The section of point b is the boundary between the two sliding paths. Energy lines A and B from point b at the inclination of *φ*_*2a*_ or *φ*_*2b*_ to the terrace surface are drawn to obtain points d and e. According to formula (6), the sliding distances *L*_*d1*_ and *L*_*d2*_ on the terrace under the sliding modes of A and B can be obtained by using the parameters *φ*_*1*_, *φ*_*2a*_, *φ*_*2b*_, *H*′_*max*_ and *L*_*s*_

6 $$\begin{gathered} L_{{d1}} = \frac{{H_{{max}}^{\prime} - tan\;\varphi _{1} L_{s} }}{{\tan \varphi _{{2a}} }} \hfill \\ L_{{d2}} = \frac{{H_{{max}}^{\prime} - tan\;\varphi _{1} L_{s} }}{{\tan \varphi _{{2b}} }} \hfill \\ \end{gathered}$$

where *H*′_*max*_ is the height of the sliding surface; *L*_*s*_ is the projection distance of the sliding surface in the horizontal direction; and *L*_*d1*_ and *L*_*d2*_ are the sliding distances on the terrace under sliding modes A and B, respectively.

As shown in the figure, since the apparent friction angle of the LST (*φ*_*2b*_) is smaller than that of the LDT (*φ*_*2a*_), the sliding distance obtained from the energy line drawn by the apparent friction angle of the LST (mode B) is larger than that determined by the apparent friction angle of the LDT (mode A).

Figures 10 and 11 show that the apparent friction angle *φ*_*2a*_ of the LDT increases with increasing shear rate, while the apparent friction angle *φ*_*2b*_ of the LST decreases with increasing shear rate. Therefore, for sliding mode A, the energy line will move towards the toe of the slope with the increase in the shear rate; that is, the sliding distance will shorten with the increase in the sliding (shear) rate. For sliding mode B, the energy line will move towards the sliding direction with the increase in the shear rate; that is, the sliding distance will increase with the increase in the sliding rate (as shown by the dotted line in Fig. 14).

Based on the above method, the sliding distances of 13 typical landslides on the South Jingyang Tableland area are calculated by the sled model, and the sliding distances calculated under different modes and shear rates are compared with the actual sliding distances. The characteristic parameters of landslides are shown in Table 3. Through this comparison, it is found that there is a great difference between the sliding distances on the terrace calculated in sliding mode A and mode B. The calculated sliding distance of mode B (*L*_*d2*_) is far larger than that of mode A (*L*_*d1*_) at different shear rates.

**Table 3:** The characteristic parameters of landslides.

No	Position	Volume (× 10^3^ m^3^)	*D*_*d*_ (m)	*H*^′^_*max*_ (m)	*L*^′^_*max*_ (m)	*φ*_*a*_(°)	*Ls* (m)	*L*_*d*_ (m)	*L*_*d1*_ (m) of Mode A			*L*_*d2*_ (m) of Mode B			Liquefaction (Yes or No)	*A*_*i*_ (^°^)
									Shear rate (cm/s)			Shear rate (cm/s)				
									0.01	0.1	1.0	0.01	0.1	1.0		
1	N34° 29′ 06″, E108° 52′ 12″	171.0	6.0	41.0	212.6	10.9	46.0	166.6	37.2	24.8	18.4	134.0	144.1	194.2	Yes	41.7
2	N34° 29′ 33″, E108° 50′ 17″	475.4	4.7	80.7	367.0	12.4	78.0	289.0	80.2	51.8	37.9	251.5	271.8	362.4	Yes	46.0
3	N34° 29′ 52″, E108° 46′ 28″	190.4	8.5	48.7	257.4	10.7	55.3	202.1	57.0	36.0	28.2	227.0	258.3	352.3	Yes	41.4
4	N34° 30′ 03″, E108° 45′ 48″	355.0	14.0	63.0	288.0	12.3	94.7	193.3	31.6	19.1	16.2	166.0	206.0	300.0	Yes	33.6
5	N34° 29′ 18″, E108° 51′ 22″	1236.0	11.5	70.4	368.8	10.8	90.1	278.7	72.7	45.2	36.9	346.0	406.5	580.0	Yes	38.0
6	N34° 29′ 30″, E108° 48′ 30″	1604.0	17.7	79.9	419.0	10.8	54.0	365.0	271.4	163.8	146.6	1900.0	2500.0	3958.3	No	56.0
7	N34° 29′ 07″, E108° 52′ 04″	308.7	19.3	62.4	169.2	20.2	59.7	109.5	155.8	94.3	85.2	1199.0	1596.0	2655.0	No	46.3
8	N34° 29′ 6″, E108° 51′ 57″	225.6	11.5	64.3	238.5	15.1	49.9	188.6	153.1	95.3	78.0	736.0	857.5	1225.0	No	52.2
9	N34° 29′ 12″, E108° 51′ 40″	94.3	8.4	64.7	164.1	21.5	32.4	131.7	201.7	126.7	99.6	797.1	894.3	1235.9	No	63.4
10	N34° 29′ 36″, E108° 47′ 41″	152.0	8.0	54.0	170.0	17.6	30.0	140.0	127.7	80.7	62.6	486.5	545.5	750.0	No	61.0
11	N34° 29′ 39″, E108° 47′ 34″	276.0	10.3	53.0	198.9	14.9	15.5	183.4	179.0	112.0	90.0	780.0	891.0	1248.0	No	73.7
12	N34° 29′ 46″, E108° 47′ 12″	430.0	14.2	56.2	275.9	11.5	18.1	257.8	228.3	139.9	119.5	1291.4	1558.6	2306.1	No	72.1
13	N34° 29′ 46″, E108° 47′ 00″	415.0	12.7	55.3	145.6	20.8	25.7	119.9	190.0	117.6	97.6	975.6	1142.9	1666.0	No	65.0

*D*_*d*_, accumulation thickness of landslide mass; *H*^′^_*max*_, height of sliding surface; *L*^′^_*max*_, maximum horizontal sliding distance; *φ*_*a*_, apparent friction angle of the sliding body; *L*_*s*_, projection distance of the sliding surface in the horizontal direction; *L*_*d*_, actual sliding distance on the terrace; *L*_*d1*_, calculated sliding distance of mode A; *L*_*d2*_, calculated sliding distance of mode B; A_i_, average inclination angle of the sliding surface.

Volume is the volume of landslide mass.

According to the comparison results between the actual sliding distance on the terrace (L_d_) and the calculated sliding distance under mode A and mode B (*L*_*d1*_*, L*_*d2*_), the loess landslides on the South Jingyang Tableland can be divided into two types (as shown in Table 3). The actual sliding distances of the No. 1–5 landslides on the terrace are 166.6–289 m, and the calculated sliding distances of mode A are 16–80.2 m under different shear rates. The corresponding calculated sliding distances of mode B are 134–586 m. The actual sliding distance of this type of landslide is closer to the sliding distance determined under sliding mode B and much longer than the calculated sliding distance determined under mode A. Therefore, it is judged that the sliding of this type of landslide is due to contact sliding between the loess sliding mass and saturated terrace soil; that is, sliding related to sliding liquefaction. However, the actual sliding distances of landslide No. 6–13 range from 109.5 to 365 m, which are close to the sliding distances of 62.5–228 m calculated by sliding mode A but much shorter than those calculated by mode B (486.5–5333 m). Therefore, it is judged that the sliding of these landslides is related to loess sliding along the dry soil layer on the terrace surface.

According to the calculated results, loess landslides on the South Jingyang Tableland can undergo not only long-distance sliding under the condition of possible sliding liquefaction (the maximum sliding distance is 289) but also long-distance sliding when the loess-sliding body slides along the terrace surface (the maximum sliding distance is 365). What is the reason for the different modes of long-distance sliding of loess landslides in this area? According to statistics, the reason for these differences in the sliding modes may be related to the variation in the average inclination angles of the sliding surfaces. The average inclination angle of the sliding surface *A*_*i*_ is defined as:

7 $${A}_{i}={\mathrm{tan}}^{-1}\left({H}_{max}^{^{\prime}}/{L}_{s}\right)$$

Through comparison, it is found that when *A*_*i*_ is less than 46°, sliding is induced by sliding liquefaction under mode B; however, when *A*_*i*_ is greater than 46°, sliding occurs under mode A along the terrace surface (see Table 3). It seems that the causes for the different types of long-distance slides are directly related to the inclination degree of the sliding surface.

It can be supposed that the reason for the different sliding modes of the loess landslides on the South Jingyang Tableland is that when *A*_*i*_ is less than 46°, the effect of the sliding mass on the terrace stratum is mainly one of horizontal pushing and scraping. The sliding body penetrates the upper dry terrace soil layer and directly contacts the lower saturated terrace soil layer, resulting in the long-distance sliding of mode B. When *A*_*i*_ is more than 46°, the effect of the sliding mass on the terrace is mainly one of vertical loading. In this case, the sliding mass does not break through the upper terrace soil layer but instead slides along the terrace surface.

In addition, for the same sliding mode, the sliding distance on the terrace is related to the sliding speed. In mode A, the faster the sliding speed on the terrace is, the shorter the sliding distance. For sliding mode B, the faster the sliding speed on the terrace is, the longer the sliding distance. Therefore, it can be concluded that the sliding mode of a landslide is related to the inclination angle of the sliding surface, while the sliding distance on the terrace is related to the sliding speed, and the changing trend in sliding distance with sliding speed is opposite under modes A and B. If the possible crack position of the sliding surface at the back edge of the slope top can be determined before the landslide occurs, the possible sliding mode of a loess landslide can be defined according to the inclination angle of the sliding surface, and the sliding distance of the landslide can be predicted according to the height of the sliding surface and the apparent friction angles along the sliding path.

## Conclusion

The results of this study are as follows.

1. Sliding liquefaction is not the only reason for the long-distance sliding of loess landslides. There are two long-runout sliding modes of loess landslides on the South Jingyang Tableland: Mode A: sliding along the terrace surface; and Mode B: sliding along a saturated terrace stratum associated with sliding liquefaction. Under these two sliding modes, loess landslides can slide long distances.
2. The sliding mode is directly related to the average inclination angle of the sliding surface and the shear interaction between different soils along the sliding path. When the average inclination angle of the sliding surface is greater than 46°, the landslide mainly slides along the terrace surface; when the average inclination angle of the sliding surface is less than 46°, the landslide mainly slides along the saturated soil layer within the terrace. The cause for the different sliding modes is related to the different interactions between the loess-sliding mass and the terrace stratum under the different inclination angles of the sliding surface. According to the average inclination angle of the sliding surface, the sliding mode of a landslide on the South Jingyang Tableland area can be distinguished.
3. Under the two sliding modes, the large shear mechanical properties of the two-layer soil composed of loess and terrace soil with different water contents show significant differences. The friction between the loess and dry terrace soil increases with increasing normal stress and shear rate, while that between the loess and saturated terrace soil presents the opposite trend. This leads to opposite trends of sliding distance with increasing sliding speed under different sliding modes. In mode A, sliding distance on the terrace decreases with increasing sliding speed; in mode B (sliding liquefaction), sliding distance on the terrace increases with increasing sliding speed.
4. The ring shear test results and the shape characteristics of the sliding surface can be combined with the sled model to identify and predict the sliding modes and sliding distances of loess landslides.
